# Case report: Two heterozygous pathogenic variants of CYP24A1: A novel cause of hypercalcemia and nephrocalcinosis in adulthood

**DOI:** 10.3389/fmed.2022.1020096

**Published:** 2023-01-10

**Authors:** Ludmila Brunerova, Ondrej Remes, Veronika Zoubkova, Pavel Votypka

**Affiliations:** ^1^Department of Internal Medicine, Faculty Hospital Kralovske Vinohrady and 3rd Faculty of Medicine, Charles University, Prague, Czechia; ^2^Nefromed, Prague, Czechia; ^3^Department of Biology and Medical Genetics, 2nd Faculty of Medicine, Charles University and Motol University Hospital, Prague, Czechia

**Keywords:** CYP24A1 mutation, hypercalcemia, nephrocalcinosis, adult, low PTH

## Abstract

**Background and aims:**

Vitamin D 24-hydroxylase is an enzyme encoded by the CYP24A1 gene, which inhibits the activation of vitamin D to form inactive metabolites. More than 20 currently described pathogenic variants (usually biallelic) of this gene are responsible for idiopathic infantile hypercalcemia manifested typically in childhood (often in newborns) with hypercalcemia, hypercalciuria, and nephrocalcinosis. However, a few patients (mostly with monoallelic heterozygous pathogenic variants) can develop mild symptoms in adulthood.

**Case description:**

We present the case of a 43-year-old male patient with hypertension and heterozygous Leiden mutation, with mural thrombi in the common iliac artery, who was sent by a nephrologist to endocrinological examination due to hypoparathyroidism, progressive hypercalcemia, hypercalciuria, and CKDG2A1. Complete laboratory and imaging methods (including PET-CT) excluded PTH-related peptide-mediated hypercalcemia and granulomatosis. Finally, the genetic analysis of the CYP24A1 gene revealed the presence of a novel combination of two heterozygous pathogenic variants: CYP24A1: c. 443T>C p.(Leu148Pro) and c.1186C>T p.(Arg396Trp).

**Conclusion:**

Differential diagnosis of patients with hypercalciuria, nephrocalcinosis, and hypercalcemia related to vitamin D exposure should include the CYP24A1 gene mutation. To the best of our knowledge, this is the first case of the novel combination of two heterozygous pathogenic variants of CYP24A1.

## Introduction

Calcium–phosphate metabolism is regulated by three key hormones—active vitamin D (1,25OHD), parathormone (PTH), and calcitonin—acting on three major players in calcium–phosphate homeostasis (kidney, bone, and small intestine) (1). Hypercalcemia represents a potentially life-threatening condition with a wide differential diagnosis. The causes of hypercalcemia comprise of two major groups: PTH-mediated (primary or tertiary hyperparathyroidism) and non-PTH mediated. Non-PTH mediated hypercalcemia includes PTH-related peptide-associated hypercalcemia (in malignancies), vitamin D-associated hypercalcemia (in benign and malignant granulomatous diseases), medication-associated hypercalcemia (associated with lithium, thiazides, etc.), hypercalcemia associated with other endocrine diseases (e.g., hyperthyroidism), hypercalcemia due to genetic causes (typically familial hypocalciuric hypercalcemia caused by mutation of the calcium-sensing receptor), and others (e.g., immobilization with high bone turnover) (2).

A minority of genetic causes is represented by mutations of the CYP24A1 (cytochrome-P450 family 24 subfamily A member 1) gene. In 2011, Schlingmann et al. identified CYP24A1 as a candidate gene for autosomal recessive infantile hypercalcemia (IHH, OMIM 143880). Loss-of-function mutations of CYP24A1 result in hypercalcemia after vitamin D exposure (3). The CYP24A1 gene encodes the mitochondrial inner membrane P450 24-hydroxylase enzyme, which inhibits the activation of vitamin D metabolites (25-hydroxyvitamin D/25OHD and 1α,25-dihydroxyvitamin D/1,25OHD) to the inactive or less active ones (to 24,25-dihydroxyvitamin D/24,25OHD and 1α,24,25 trihydroxyvitamin D/1,24,25OHD) (3–5). The phenotype of IIH includes a wide spectrum of clinical scenarios (6), from severe forms, diagnosed early in infancy [severe hypercalcemia manifested as failure to thrive, dehydration, vomiting, and nephrocalcinosis (7)] to milder forms, often diagnosed in adulthood during a workout for recurrent nephrolithiasis (8). However, most patients with CYP24A1 mutation (particularly monoallelic heterozygous) remain unrecognized, as only a few hundred cases have been reported up to date (9, 10).

Herein, we present the case of an adult male patient with hypercalciuria, nephrocalcinosis, and intermittent progressive hypercalcemia caused by two heterozygous pathogenic variants of CYP24A1. The patient agreed with the anonymous presentation of his case, and he provided signed informed consent.

## Case presentation

A 43-year-old male patient with marfanoid habitus (190 cm, 73 kg with long extremities) has been treated for hypertension since 2013 (ramipril 5 mg daily and felodipine 5 mg daily). Ultrasonography revealed nephrocalcinosis, and a CT scan described atherosclerotic aorta and common iliac arteries with mural thrombi. A geneticist excluded Marfan syndrome and found a heterozygous factor V. (Leiden) mutation. A cardiologist started treatment with aspirin 100 mg daily and atorvastatin 10 mg daily. A nephrologist diagnosed slightly decreased renal function (CKDG2) with normal albumin/creatinine (ACR) and protein/creatinine (PCR) ratio, normal urine analysis, and hypercalciuria and initiated the treatment with hydrochlorothiazide (12.5 mg daily). The patient was referred to an endocrinologist for possible hypoparathyroidism (serum calcium levels at the lower limit of the normal range and low PTH) in 2019.

The patient denied any history of renal stones and fractures. His father had hypertension and died of colorectal cancer, and his mother, siblings, and two children were healthy. There was no history of nephrolithiasis in the family.

At the endocrinological examination, the physical finding (except for marfanoid habitus) was normal, and no bone abnormalities were observed. The laboratory test revealed hypoparathyroidism but did not confirm hypocalcemia (serum calcium/S-Ca 2.50 mmol/l). Genetic analysis of the calcium-sensing receptor (Ca-SR) gene, performed due to low PTH and initially lower serum calcium, did not find any pathogenic variants.

Serum calcium levels progressively increased during the follow-up, and thus, hydrochlorothiazide was stopped in 2020, with practically no effect on calcium levels. The patient kept visiting both his nephrologist and his endocrinologist, however, very irregularly, and he missed his appointment several times; thus, the parameters were quite sparse (in overview in Table 1).

**Table 1:** Laboratory finding throughout the time of follow-up.

**Parameter/** **year**	**2018**	**2019**	**2020**	**2021**	**2022**
S-Ca (2.2–2.6 mmol/l)	2.2–2.5–2.66	2.69	2.63	2.8–3	2.69
S-Ca-i (1.0–1.4 mmol/l)	x–x−1.28				
S-P (0.7–1.5 mmol/l)	0.88–x−0.9	1.26		0.9–x	0.88
S-Mg (0.7–1.0 mmol/l)	0.69–x−0.8	0.77		0.88–x	
PTH (1.6–6.9 pmol/l)	0.58–x−0.3	0.47		0.3–x	
25OHD (75–150 nmol/l)	85–x−141	135	75.4	141–x	
1.25OHD (19.9–79.3 ng/l)	x	61		67–x	
S-crea (64–104 μmol/l)	102–115–126	134	137	136–x	135
DU-Ca (2–5 mmol/day)	8.5–x–x	6.1		6.1–7.7	5.5

The data comes from both—the nephrological and the endocrinological examinations.

S-Ca, serum calcium; S-Ca-i, serum ionized calcium; S-P, serum phosphate; S-Mg, serum magnesium; PTH, parathormon; 25OHD, 25-hydroxyvitamin D; 1,25OHD, 1,25-dihydroxyvitamin D; S-crea, serum creatinin; DU-Ca, total amount of urine calcium/24 h; x, unmeasured parameter at certain time point.

The highest level of serum calcium (3 mmol/l) was observed during the summer of 2021. In between, the detailed work-up was carried out for the exclusion of PTH-related peptide-associated hypercalcemia and granulomatosis-associated hypercalcemia. Complete laboratory tests showed normal serum protein electrophoresis including immunofixation, normal serum level of ACE/angiotensin-converting enzyme, and normal level of 1,25OHD. The markers of bone turnover were within the normal range (cross laps 331 ng/l: normal range 182–801; and P1NP 27.6 μg/l: normal range 15–80).

There were normal findings in densitometry (Z-score of lumbar spine 1.1; Z-score of proximal femur −0.1 and trabecular bone score 1.485), and ^99m^Tc/technetium scintigraphy and ^18^FDG-PET/CT (fluorodeoxyglucose positron emission tomography/computed tomography) did not reveal any pathology.

In the end, in January 2022, the genetic examination for CYP24A1 was performed by massively parallel sequencing using a Clinical Exome Solution (CES, Sophia Genetics, Switzerland) comprising ~5,000 Mendelian disease-related genes. DNA library was sequenced on the NextSeq platform (Illumina, USA). Sequencing data were processed and analyzed by Sophia DDM software. Variant prioritization was also performed by Sophia DDM software and supported by Integrative Genomics Viewer (IGV; Broad Institute), Alamut Visual (Interactive Biosoftware), and VarSome Clinical software. Variant prioritization was carried out on the basis of frequency in the general population (gnomAD and dbSNP databases), presence and frequency in clinical databases (i.e., ClinVar), interspecies conservation of the residue and coherence, and *in silico* predictions using tools integrated into VarSome Clinical software (DANN, DEOGEN2, EIGEN, FATHMM-MKL, M-CAP, MVP, MutationAssessor, MutationTaster, PrimateAI, REVEL, PolyPhen, and SIFT). The pathogenicity of the detected variants was classified into five categories according to the evidence criteria proposed by the American College of Medical Genetics and Genomics (ACMG) and the Association for Molecular Pathology (AMP) guidelines (11). We identified two pathogenic variants in the CYP24A1 gene: NM_000782.5(CYP24A1):c.443T>C p.(Leu148Pro) and NM_000782.5(CYP24A1):c.1186C>T p.(Arg396Trp). The ACMG classification of these pathogenic variants is as follows: NM_000782.5(CYP24A1):c.443T>C p.(Leu148Pro)—class 5 (PM2, PP2, PP3, PP5, and PS3) and NM_000782.5(CYP24A1):c.1186C>T p.(Arg396Trp)—class 5 (PM2, PP2, PP3, PP5, and PM5). Detected variants were validated by Sanger DNA sequencing.

We strongly recommended the genetic analysis of the patient's relatives; however, neither of them has undergone the blood sampling yet. Nonetheless, we advised the patient to inform his relatives that vitamin D supplementation and excessive calcium intake could be risky in terms of developing severe hypercalcemia if CYP24A1 mutation is present.

The patient was instructed to maintain higher fluid intake, not to be expose to vitamin D (including sun exposure if possible), and to avoid excessive calcium intake. During further follow-up, calcium levels remained stable and slightly above the upper normal range.

## Discussion

Loss-of-function mutations of the CYP24A1 gene lead to increased levels of active metabolites of vitamin D causing hyperabsorptive hypercalcemia, hypercalciuria, nephrocalcinosis, and nephrolithiasis, typically diagnosed in childhood as IHH. Although rare in their occurrence (12), they should be considered as the cause of hypercalcemia, particularly if associated with nephrocalcinosis and/or nephrolithiasis, even in adults. In our patient, the laboratory findings (hypercalcemia, hypercalciuria, and suppressed PTH levels) were typical for the carriers of loss-of-function mutations in CYP24A1. However, normal levels of 1α,25OHD and negative family history did not fit into the typical clinical picture; thus, we first focused on the exclusion of other (more common) causes of hypercalcemia with suppressed PTH levels. The levels of 1α,25OHD were measured two times during the follow-up and were surprisingly at the upper limit of the normal range but not elevated as would be expected in the case of CYP24A1 loss-of-function mutations. However, the heterogeneity of 1,25OH levels is very high in patients with mild forms of IHH (and their adult relatives), ranging from near-normal levels to highly elevated ones (13). Furthermore, in the Molin's et al. study (14) of patients with hypersensitivity to vitamin D, the levels of 1,25OHD did not significantly differ among patients with or without CYP24A1 mutation. We can just speculate that the combination of these pathogenic variants is not necessarily associated with elevated levels of 1,25OHD (although no functional studies were done to confirm this hypothesis and since it is the first described case of this combination of pathogenic variants, no literature data are available).

During the sequencing, two pathogenic variants in the CYP24A1 gene were identified, NM_000782.5(CYP24A1):c.443T>C p.(Leu148Pro) and NM_000782.5(CYP24A1):c.1186C>T p.(Arg396Trp), and validated by Sanger DNA sequencing. The former is known as missense mutation resulting in a 25–50% decrease in the activity of CYP24A1 (15–17). The latter is also a missense mutation (18–25); however, to the best of our knowledge, no case of the common occurrence of two heterozygous pathogenic variants, p.(Leu148Pro) and p.(Arg396Trp), has been described yet.

The blood samples of our patient were analyzed repeatedly throughout the year with the highest levels of calcium found during the summer period (the patient was an active football player massively exposed to sunlight). The fluctuations of serum calcium levels related to sun exposure in the p.(Arg396Trp) mutation carriers were described previously (21, 24).

Bone densitometry found normal age- and gender-related BMD, and markers of bone turnover were also normal. Similar findings were observed in a small study of heterozygous (Arg396Trp) mutation carriers, which did not find any major differences in bone health if compared with the wild type (23). However, in preclinical studies, mice with CYP24A1 mutations (complete deficiency of the enzyme) revealed impaired intramembranous bone mineralization due to the accumulation of osteoid caused by elevated 1,25OHD levels (26). No data on bone health in p.(Leu148Pro) mutation carriers have (to the best of our knowledge) been described yet. Based on our case, we suggest that the carrier of two heterozygous pathogenic variants, p.(Leu148Pro) and p.(Arg396Trp), does not seem to have a negative impact on bone health (although it could be modified by intensive physical activity of our patient).

In terms of non-pharmacologic treatment, it is reasonable to avoid exogenous vitamin D supplementation and excessive sunlight exposure; however, the efficacy of these approaches has to be confirmed (9). In our patient, this approach seemed to be quite effective (particularly the avoidance of un-protective sunlight exposure).

Hypercalcemia can be managed with intravenous 0.9% saline and frusemide, but steroids are not effective since functional CYP24A1 is required for its action (27, 28). Other therapeutic options described in the literature include the use of azoles (reducing the synthesis of 1,25OHD by inhibiting CYP27B1) (29–31), rifampin (inducer of CYP3A4 enzyme that catalyzes non-specific hydroxylation of 1,25OHD to inactive metabolite) (32), supplementation with L-methionine for urinary acidification as the prevention of calcium stone formation (33), or bisphosphonates in severe cases of IHH (25).

No association between the factor V. Leiden mutation and the mutations pertaining to vitamin D metabolism was found in the literature.

It would be proper to perform a segregation analysis of the proband's parents in order to confirm the variants being on both the alleles—that is, trans-heterozygous (as considered); however, his father is unavailable, and his mother and siblings have not been willing to undergo the blood sampling yet.

## Conclusion

Biallelic homozygous mutations of the CYP24A1 gene lead to the typical phenotype of IHH; however, even two heterozygous pathogenic variants can result in hypercalcemia, hypercalciuria, and nephrocalcinosis/nephrolithiasis, but due to relatively mild symptoms, it can be diagnosed (in comparison with IHH) late in adulthood. In the presented case of a young male, the novel common occurrence of two heterozygous pathogenic variants p.(Leu148Pro) and p.(Arg396Trp) has been identified. Genetic analysis of CYP24A1 should be considered in patients with hypercalcemia and suppressed PTH, particularly if associated with hypercalciuria and nephrocalcinosis/nephrolithiasis, despite normal levels of 1,25OHD.

## Data availability statement

The datasets presented in this article are not readily available to protect patient privacy and confidentiality. Requests to access the datasets should be directed to the corresponding author(s).

## Ethics statement

Ethical review and approval was not required for the study on human participants in accordance with the local legislation and institutional requirements. The patients/participants provided their written informed consent to participate in this study. Written informed consent was obtained from the individual(s) for the publication of any potentially identifiable images or data included in this article.

## Author contributions

LB prepared the manuscript. OR, VZ, and PV revised the manuscript. All the authors contributed in the clinical and genetic diagnosis of the patient. All authors contributed to the article and approved the submitted version.
